# "Hugh Woods, M.D.," and the Newspapers

**Published:** 1890-02-22

**Authors:** Hugh Woods

**Affiliations:** Highgate, N.


### Editor's Letter-Box.

[Our Correspondents are reminded that prolixity is a great bar to publication, and that brevity of style and conciseness of statement greatly facilitate early insertion.]

"HUGH WOODS, M.D.," AND THE NEWSPAPERS.

As a grateful acknowledgment of the amusement afforded to myself and my friends by "Agricola" in The Hospital of February 15th, I think I must condescend to devote a little of my leisure time to the task of answering him according to his folly. This "wilful and mischievous child," who writes "in a style redolent of the nursery" (though this is hardly "precocious" in a man of thirty, unless there is something wrong with modern nurseries), is naturally anxious to know when the "sharp and well-merited castigation," with which he is threatened, is to come off, and what is to be the instrument of torture. "Agricola" should get those careless nursery maids, the Editors, dismissed, for letting me get into such mischief. It is hardly likely that they would box his ears, if he tries it on. Does "Agricola" style himself so in allusion to his boorishness. "Agricola" compares me to a naughty child. I would compare him to a clownish bully, who becomes very valiant when he is sure that his antagonist will not strike back. Of all the letters I have written, why challenge that one alone as to which I said from the first that I would not give my authority? Why not answer my letter to the *Times* of December 30th, or other letters to the *Echo*, or recent letters to the *Star*, since the *Echo* suddenly changed its attitude towards me? Is it not manly to attack Editors for publishing letters that "good-humoured indulgence" allows to pass unrefuted? Is it possible that no one can find leisure to test the accuracy of my "profound knowledge of metropolitan hospitals," the rapid acquirement of which seems to stagger "Agricola"? As he considers me a "precocious pretender," he can put it down either to "precocity," or "pretence," or to both, as he pleases.

Trusting that The Hospital has not made the resolve to decline publishing my writings, which "Agricola" recommends, to his own advantage, no doubt, I am, Sir, yours faithfully, Hugh Woods, M.D.

Highgate, N.

[The product of Dr. Hugh Woods' gratitude, amusement and condescension proves acrid enough to set us wondering what a combination of less amiable qualities might mean in his case. The certificate of his age is unnecessary. Whether he be thirty or ninety, he has yet to afford some better justification for running amuck among the Hospitals of London than is supplied by a diploma recently acquired in Ireland, and which apparently gives the measure of his experience, or the unsupported statements of his anonymous friend "who is unconnected with hospitals or the medical profession." Dr. Hugh Woods has made the very gravest charges against hospitals and hospital workers, and appears incapable of understanding that the *onus probandi* rests upon him and that an honourable man would feel bound either to substantiate or withdraw his indictments. Doubtless, Dr. Woods has persuaded himself that anyone who tells him this is a "clownish bully," but neither the exuberant grace of his language nor its misdirected force is likely to divert attention from the real issue.—Agricola.]

